# Quality assessment of fluoroscopic imaging obtained during neonatal contrast enema exams

**DOI:** 10.1007/s00261-026-05400-9

**Published:** 2026-03-31

**Authors:** Shyam Sunder B. Venkatakrishna, Levy C. Onyango, Carmen Rosa Cerron-Vela, Devyn C. Rigsby, Mohammad Jalloul, Dana Alkhulaifat, Hansel J. Otero, Savvas Andronikou

**Affiliations:** 1 Department of Radiology, Children’s Hospital of Philadelphia, Philadelphia, USA; 2 Department of Radiology, Perelman School of Medicine, University of Pennsylvania, Philadelphia, USA

**Keywords:** Contrast enema, Fluoroscopy, Neonate, Quality assessment

## Abstract

**Purpose:**

The American College of Radiology (ACR) and Society for Pediatric Radiology (SPR) have published practice parameters for pediatric fluoroscopic contrast enema (CE) exams. The purpose of our quality assurance (QA) study was to assess adherence to neonatal (< 2 days of age) CE practice parameters at a large tertiary care pediatric hospital.

**Methods:**

Image database review identified all CE exams performed on children ≤ 2 days old at our institution between February 2019 and August 2022. Two pediatric radiologists conducted independent review of CE imaging and reports for six subjective quality criteria. One radiologist assessed six objective quality metrics. Data were summarized as counts and percentages and inter-reviewer agreement *p*_o_ was calculated for the subjective metrics.

**Results:**

70 neonatal CEs met inclusion criteria and were reviewed. For subjective criteria: A small-caliber rectal catheter was used in 93% (R1) to 94% (R2) of cases. 96–97% of exams included a true lateral rectal view; this view was obtained at early filling in 83–86% of cases. The rectosigmoid index was readily assessed in nearly all studies (100% R1; 97% R2). The entirety of the colon was visualized through to the cecum in 87% of studies. In 74–76% of cases, the appendix and/or terminal ileum were visualized. Observed inter-reader agreement was substantial for all metrics, ranging from 83% to 97%. For the objective quality metrics: 83% of CEs included a scout image. Lateral rectal imaging included visualization of the sacrum in 91% of cases. Radiation dose and fluoroscopy time were documented in 90% of cases. 63% of cases were performed without direct exposures. All 70 CEs were performed without documented complications.

**Conclusion:**

Although the majority of studies included a scout image, radiation dose and fluoroscopy time, operators should be reminded to collect and store these basic components for every exam. Similarly, while most cases used an appropriately small rectal catheter, staff should be aware to do so in every exam. Approximately one-fourth of studies did not include visualization of the appendix and/or terminal ileum, which may reflect the difficulty of obtaining these views for certain pathologic entities in neonatal practice. Our study demonstrates the importance of continuous quality assessment even in expert centers and we recommend that a Quality Standards checklist be used for all neonatal CE studies, possibly incorporated as part of a template report.

## Introduction

Neonatal contrast enema (CE) is a common fluoroscopic exam performed by pediatric radiologists for suspected abnormalities in the colon, rectum or distal small intestine [1–4]. It is the preferred examination for suspected neonatal low intestinal obstruction, having a high diagnostic efficacy [5, 6]. In the emergency setting, contrast enema can aid in distinguishing various conditions (some of which require surgery) and can be therapeutic in conditions such as uncomplicated meconium ileus. Radiation exposure remains a concern in children [7, 8] and in accordance with ALARA (as low as reasonably achievable), pediatric radiologists continuously attempt to minimize radiation exposure while maintaining diagnostic accuracy [9–12]. In the delicate realm of neonatal care, where precision and safety are paramount, the implementation of standardized protocols in the performance and interpretation of CE is of utmost importance. By establishing clear guidelines and procedures, these protocols ensure consistency, accuracy, and safety in the administration of contrast enemas to neonates, who are particularly vulnerable to complications and adverse events. The American College of Radiology and Society for Pediatric Radiology [ACR–SPR] have published practice parameters (revised in 2021) to provide guidance for performing contrast enema examinations in children [13]. The purpose of our quality assurance (QA) study was to assess adherence to neonatal (*≤* 2days of age) CE practice parameters at a large tertiary care pediatric hospital.

## Methods

**Fig. 1:**
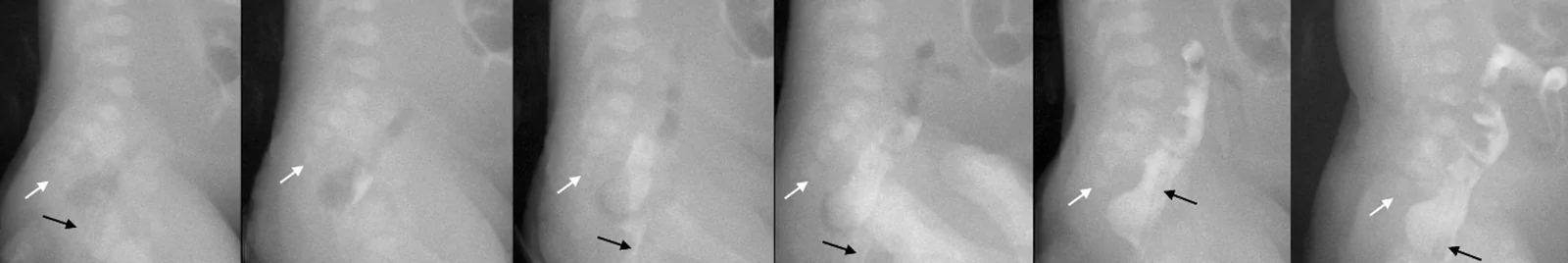
Pediatric fluoroscopic contrast enema examination meeting the quality criteria based on the ACR-SPR practice parameters in a 1-day-old boy with a distended abdomen and failure to pass meconium. The initial image demonstrates the use of a small caliber catheter (black arrow) with the patient in a true lateral position, including the sacrum (white arrow), and gentle filling of the rectum over the course of the image grabs (which are grainier than full exposures), not causing abrupt distention of the rectum and therefore allowing for comparison of rectal and sigmoid diameters

**Fig. 2:**
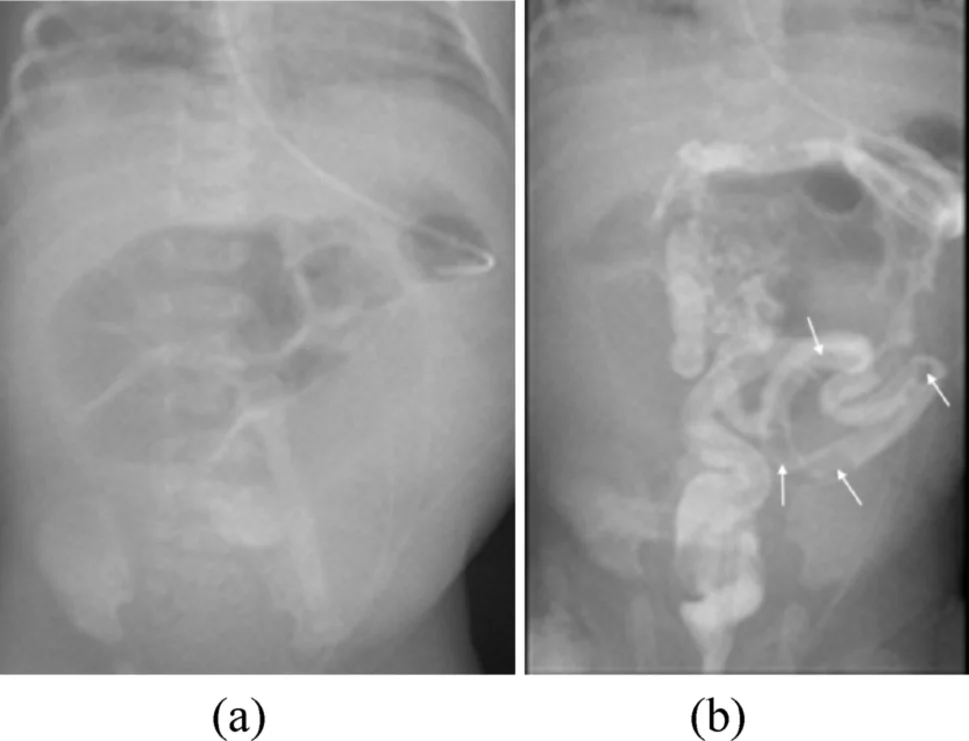
Pediatric fluoroscopic contrast enema examination meeting the quality criteria based on the ACR-SPR practice parameters in a 1-day-old girl with abdominal distention and failure to pass meconium. **a** The scout view plays a role in demonstrating the dilated central bowel loops without distal gas, confirming the low obstruction, and for excluding perforation with free air, before proceeding with a contrast enema. **b** The contrast was successfully introduced up to the level of the caecum and terminal ileum, which allowed demonstration of a microcolon, with visible colonic meconium plugs, seen as, long and sometimes continuous, filling defects (white arrows). Persistence during the examination with passage of contrast proximally into the small bowel shows a small caliber of the small bowel indicating that the obstruction is more proximal, as would be the case with small bowel atresia

**Fig. 3:**
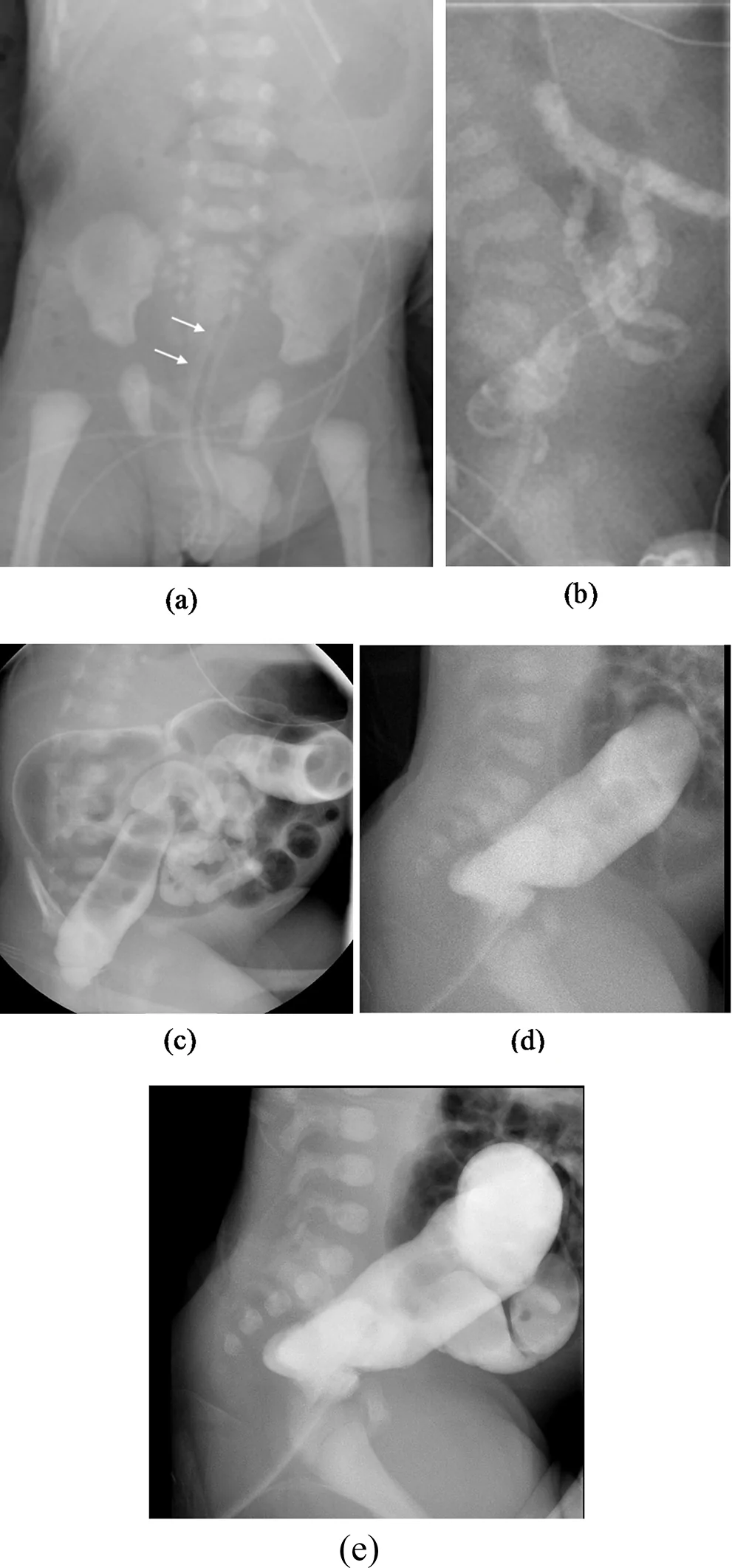
Examples of 4 different pediatric fluoroscopic contrast enema examinations NOT meeting the quality criteria based on the ACR-SPR practice parameters. **a** Large catheter: AP view in a baby boy on the first day of life, with history of intestinal obstruction diagnosed in utero and suspected jejunal atresia. A large rectal catheter has been placed in the rectum for the contrast enema examination (arrows) instead of a small caliber catheter as recommended (compare to Fig. 1). **b** Exclusion of sacrum on the lateral view: Lateral view in a baby boy on the first day of life with history of intestinal obstruction diagnosed in utero and suspected jejunal atresia. Even though a lateral view is provided at the start of the contrast enema examination, the collimation is tight, and the sacrum falls outside the field of view, which precludes evaluation for congenital anomalies, sacrococcygeal masses in the pre-sacral space, determination of whether the image is truly lateral and evaluation for surgical planning. **c** No lateral view: Oblique view in a 1-day-old boy with delayed passage of meconium does not provide any true lateral views for adequate evaluation of the rectum, the recto-sigmoid ratio and any sacral anomalies. **d** Good quality image grab of the lateral rectal filling and **e** unwarranted additional full exposure of the lateral rectum taken immediately after, with no additional diagnostic benefit

This was an IRB approved, HIPAA compliant, retrospective study done at a large, tertiary children’s hospital in the United States. A retrospective quality review of contrast enema exams performed on children ≤ 2 days of age, between February 2019 and August 2022, was conducted. The dichotomous subjective and objective quality metrics used in this study were defined based upon ACR–SPR [American College of Radiology - Society for Pediatric Radiology] practice parameter for the performance of pediatric fluoroscopic contrast enema examinations as provided in Table 1. The subjective quality review was performed by two pediatric radiologists [L.C.O. and C.R.C-V. (one year of experience)] with independent review of all contrast enema exams for adherence to 6 subjective quality metrics. The objective quality review was performed by one pediatric radiologist [L.C.O.] with the review of all contrast enema exams for adherence to 6 objective quality metrics (Table 1). Figures 1, 2 and 3 are representative example figures of pediatric fluoroscopic contrast enema examinations that meet/do not meet the quality criteria based on the ABR-SPR practice parameters.

**Table 1:** Subjective and objective quality metrics used in this study

Subjective quality metrics	Objective quality metrics
Small-caliber rectal tube used	Scout image present
True lateral rectal view obtained	Lateral rectal view includes visualization of sacrum
Lateral rectal view obtained at early filling	Radiation exposure indices documented
Rectosigmoid ratio able to be assessed	Fluoroscopy time documented
Entirety of colon visualized through to cecum	Study performed without direct exposures
Appendix and/or terminal ileum visualized	Study documented as performed without complications

### Statistical analysis

Inter-reviewer agreement was assessed for two independent raters across categorical variables. Descriptive statistics, including category frequencies and percentages, median, and interquartile range (IQR), were calculated to summarize the data. Initial Cohen’s Kappa calculated values were lower than observed agreement, reflecting the Kappa Paradox. Gwet’s Agreement Coefficient 1 (AC1) was selected as the final measure because it is less sensitive to prevalence and provides a more stable, chance-corrected agreement [14]. Agreement was interpreted using the following thresholds: values below 0.20 indicate poor agreement, 0.21–0.40 fair, 0.41–0.60 moderate, 0.61–0.80 substantial, and above 0.80 almost perfect agreement. All analyses were conducted using **Stata/SE 18.0** (StataCorp LLC, College Station, TX).

## Results

70 CEs for children ≤ 2 days old were performed during the study period (69 children [41 males], one child underwent two CEs). Indications included: abdominal distention with concern for bowel obstruction (30), delayed passage of meconium (24), bilious emesis (8), prenatally diagnosed small bowel atresia (4), suspected Hirschsprung Disease (2), omphalocele evaluation (1), and suspected intestinal malrotation (1). Diagnoses included: normal (21), microcolon (18), Hirschsprung Disease (13), small left colon syndrome (11), meconium plug syndrome (5), colonic atresia (1), and segmental small bowel atresia (1).

**Fig. 4:**
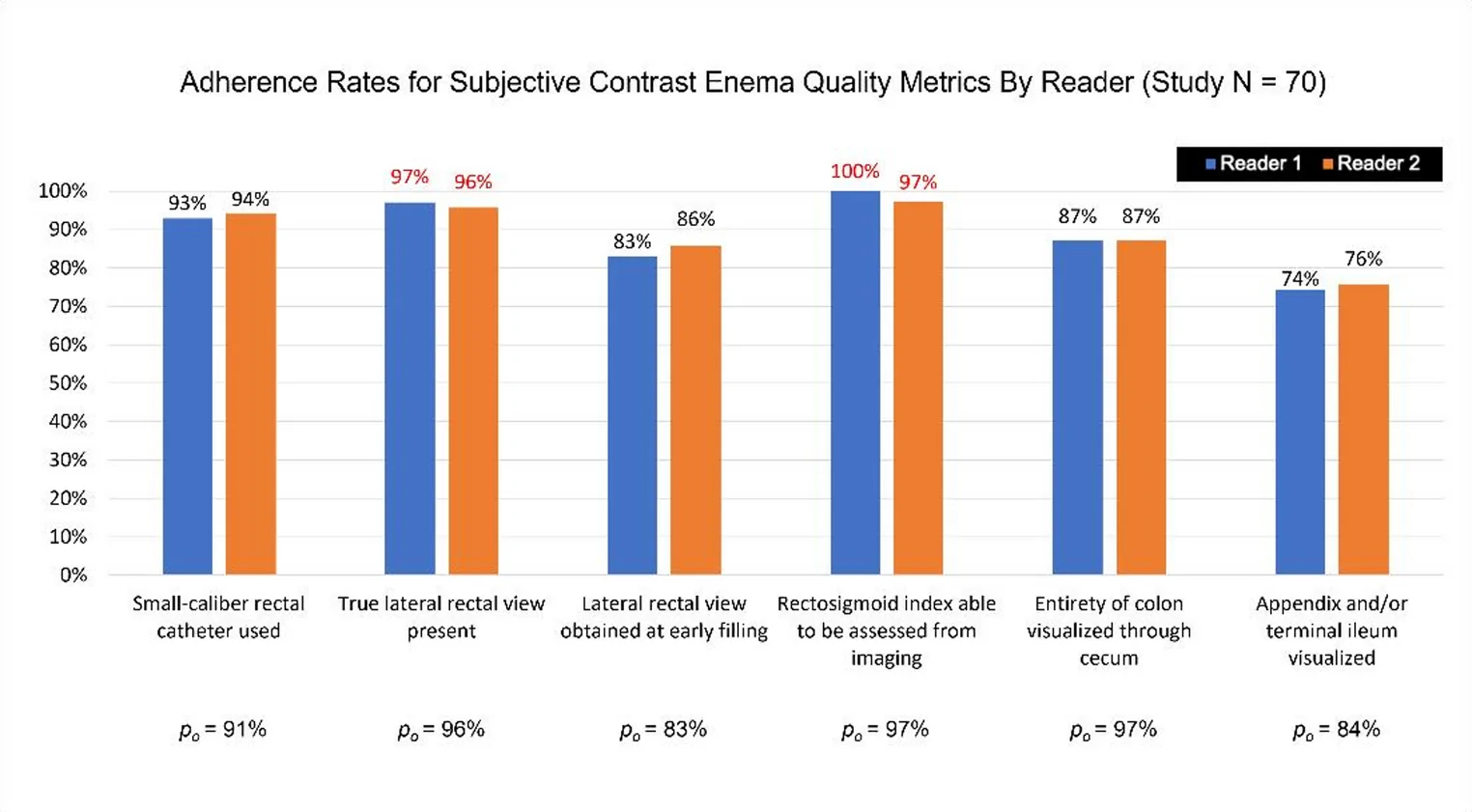
Graph representing adherence rates for subjective contrast enema quality metrics by readers 1 and 2

*Subjective metrics *(Fig. 4):

A small-caliber rectal catheter was used in 93–94% of CEs [65/70 reader 1 (R1); 65/69 reader 2 (R2); inter-reader agreement (*p*_*o*_) 91% and AC1 = 0.90 (95%CI: 0.81–0.99].

A true lateral rectal view was recorded in 96%−97% [*p*_*o*_ = 96%] and AC1 = 0.95 (95%CI: 0.88–1.00].

A lateral rectal view at early filling was recorded in 83–86% [58/70 R1; 60/70 R2; *p*_*o*_ = 83% and AC1 = 0.77 (95%CI: 0.62–0.91].

The rectosigmoid index could be assessed in 97–100% [66/66 R1; 68/70 R2; *p*_*o*_ = 97% and AC1 = 0.97 (95%CI: 0.90–1.00].

Both R1 and R2 visualized the entirety of the colon through to the cecum in 87% [61/70; *p*_*o*_ = 97% and AC1 = 0.96 (95%CI: 0.91–1.00].

The appendix and/or terminal ileum were visualized in 74–76% [52/70 R1–53/70 R2; *p*_*o*_ = 84% and AC1 = 0.75 (95%CI: 0.60–0.90].

**Fig. 5:**
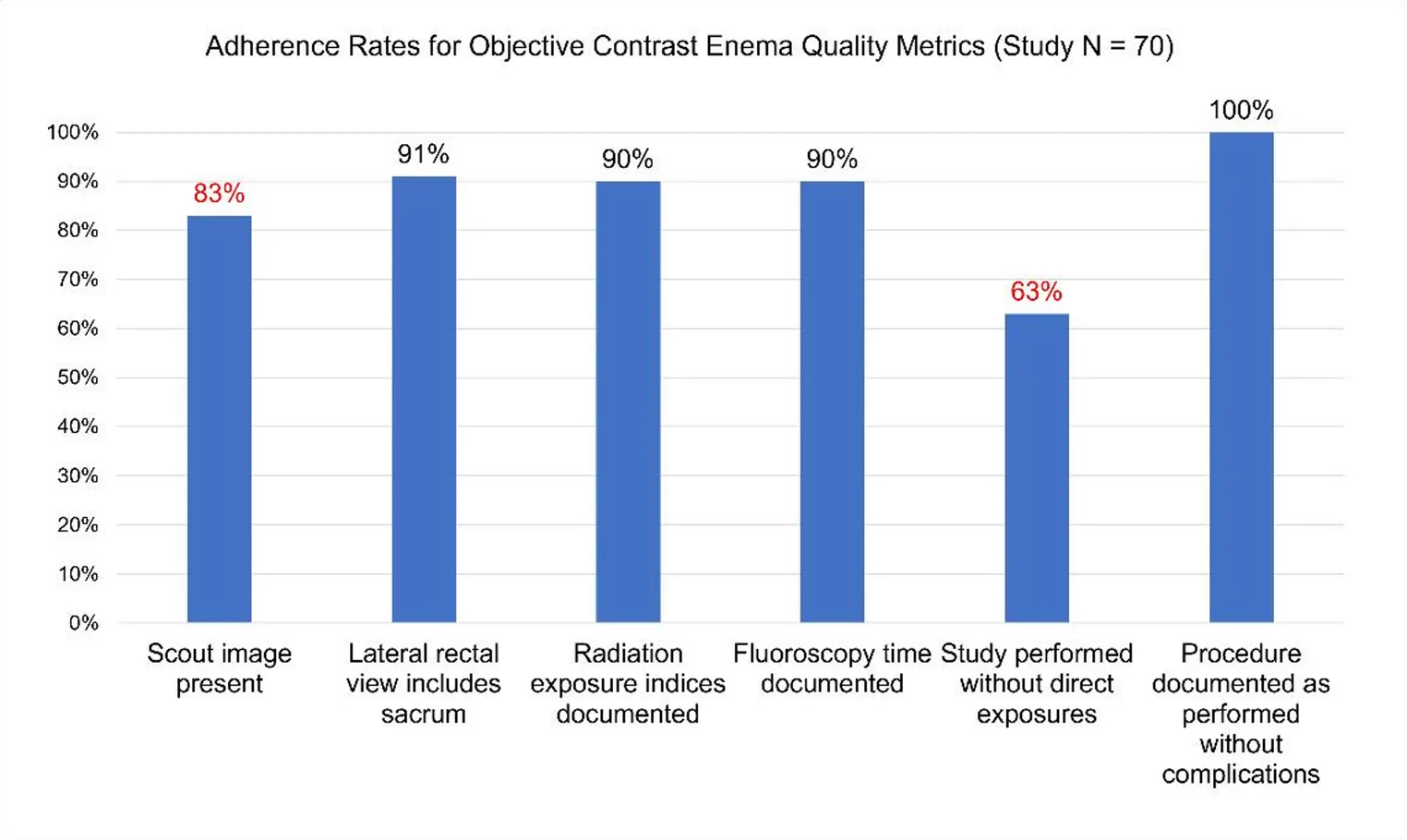
Graph representing the adherence rates for objective contrast enema quality metrics

*Objective metrics* (Fig. 5): 83% (58/70) included a scout image; in 91% (64/70), lateral rectal imaging included the sacrum; radiation dose was documented in 90% (63/70); the median dose area product was 0.5 dGy•cm^2^ (IQR 0.3–1 dGy•cm^2^); fluoroscopy time was documented in 90% (63/70); median fluoroscopy time was 1.5 min (IQR 1.1–2.5); and 63% (44/70) were performed without direct exposures. In 37% (26/70) direct exposures ranged from 1 to 7 (with a median of one). All 70 CEs were performed without documented complications. All examinations were performed with Cysto-Conray^®^ II (Iothalamate meglumine injection USP 17.2%; Liebel-Flarsheim Company LLC, Raleigh, NC, USA).

## Discussion

Subjective quality metrics had ≥ 85% adherence with high inter-reviewer agreement, except for the initial lateral rectal image acquisition not being properly timed to early filling and visualization of the appendix and/or terminal ileum. There was < 85% adherence to two objective quality criteria metrics including study performed without direct exposures (63%) and scout image present (83%). This was unexpected at a tertiary, pediatric hospital staffed exclusively by subspecialist fellowship-trained pediatric radiologists.

Radiology practice requires continuous quality assessment even at large pediatric referral centers, with subspecialist pediatric radiologists. Pediatric radiologists are trained to utilize dose-reduction features such as pulsed fluoroscopy, last-image capture, and to minimize fluoroscopy time and magnification [15, 16]. However, studies should also meet the diagnostic objectives. The objective of a CE in neonatal obstruction is to narrow down the possible causes and identify those etiologies requiring surgery [5]. The principal differential diagnoses identified through CE are Hirschsprung disease, colonic atresia, small left colon syndrome, meconium ileus and small intestinal atresia [5, 6, 17].

A recent study by Baad et al. [5] summarized and investigated the following features required for distinguishing the main pathologies sought for in neonates with low obstruction: scout view; rectosigmoid index reversal; serrations; transition zone and Microcolon (with abrupt ileal cut off vs. many ileal meconium filling defects). For identifying these important radiologic features, the CE procedure must be performed appropriately and the ACR–SPR practice parameters address these requirements [13]. The scout image is used for (a) identifying calcifications which may indicate meconium peritonitis; (b) pneumatosis, portal venous gas and pneumoperitoneum which would result in the procedure being cancelled/postponed and surgical decision making, and (c) the obstruction pattern (no obstruction, low obstruction, high obstruction) which would confirm whether the contrast enema is the appropriate test. The lateral view is integral to the diagnosis of Hirschsprung disease, and requires the sacrum be included for evaluation of patient positioning. Ensuring the spasmodic abnormal distal portion of the rectum in Hirschsprung disease is not distended out by either a large caliber rectal tube or by overfilling, requires that the rectum be imaged during early filling. These quality components of the study are required for the rectosigmoid ratio to be assessed, which has a high specificity for diagnosing Hirschsprung disease [5].

Getting contrast through the entire colon and into the terminal ileum should be a goal of all neonatal contrast enemas, noting however, that contrast will not pass through a colonic atresia by definition, and that it may not be possible to reflux contrast into the terminal ileum in up to a third of cases due to ‘*competency of the ileocecal valve*’ [5]. In addition, in instances where a transition zone is identified, further filling of dilated more proximal colon is not recommended, and the caecum will not be demonstrated. However, some of the important diagnoses in the context of neonatal contrast enema rely on showing the whole colon and distal ileum.

Finding a colonic transition zone (if the rectum is deemed normal) has a high sensitivity for diagnosing Hirschsprung disease [all the other features - recto-sigmoid index and serrations are less sensitive and more specific]. In addition, detecting a microcolon for diagnosing meconium ileus and small intestinal atresia is mandatory in neonatal CE since surgical intervention is necessary for some cases of meconium ileus and all cases of atresia [5]. A microcolon by definition is a small caliber colon throughout its length, which requires getting contrast to the caecum, and which in turn is recognized by opacifying and identifying the appendix or terminal ileum. Furthermore, once a microcolon is diagnosed, distinguishing ileal atresia (always requiring surgery) and meconium ileus (often treatable without surgery) depends on contrast be refluxed into the terminal ileum – an abrupt ileal cut off with a small caliber terminal ileum indicates small intestinal atresia while a distended terminal ileum with many meconium filling defects indicates meconium ileus.

The Pause and Pulse campaign (of the Image Gently program), is focused on strategies for optimizing the dosage in pediatric fluoroscopy [18]. Documentation of radiation exposure indices is more relevant than documentation of fluoroscopic time, but the ACR-SPR practice parameter, recommends both that fluoroscopy time be minimized and recorded [13] and where possible that other metrics relative to radiation dosage, such as dose rate, DAP (dose area product), or air kerma be recorded [13]. The radiation dose and fluoroscopy time in our study were documented in 90% (63 CEs). The ACR-SPR, is also clear in recommending the use of ‘*digital pulsed fluoroscopy*,* last image hold*,* and screen save features help to reduce radiation dose’* when possible [13]. They specifically state that screen saves are ‘*preferable to spot images or overhead radiographs*’ for minimizing radiation dose. 37% of cases in our study included at least one direct exposure (spot image). We note that exposures may be necessary according to radiologists’ discretion. When utilizing pulsed fluoroscopy and low dose techniques, there may be a tendency to capture spot films to improve visualization of detail. However, neonatal contrast enemas rely largely on identifying caliber differences and filling defects which do not require high detail but do require imaging of the whole colon and even terminal ileum where possible.

### Limitations

As this was a single-center retrospective study conducted at a large pediatric tertiary hospital, the observed image quality may not be fully representative of that achievable in smaller pediatric centers or general hospitals.

The current American College of Radiology and Society for Pediatric Radiology [ACR–SPR] practice parameters were revised in 2021 while this examinations for this study were from February 2019 to August 2022 i.e. some predate the revised practice parameters.

Two of the 6 subjective parameters, ‘*entirety of the colon visualized through to cecum*’ and ‘*appendix and/or terminal ileum visualized*’ may not be achieved because of practical considerations and are not always quality failure. As mentioned in the manuscript above, contrast will not pass through a colonic atresia by definition, and it may not be possible to reflux contrast into the terminal ileum in up to a third of cases due to ‘competency of the ileocecal valve’ [5]. In addition, in instances where a transition zone is identified, further filling of dilated more proximal colon is not recommended, and the caecum will not be demonstrated.

## Conclusion

Although the majority of studies followed quality metrics, including a scout image, radiation dose and fluoroscopy time, operators should be reminded to collect and store these basic components for every exam. Approximately one-fourth of studies did not include visualization of the appendix and/or terminal ileum, which may reflect the difficulty of getting contrast around the entire colon for certain pathologic entities in neonatal practice. Although ACR-SPR guidelines discourage direct exposures during CE exams, 37% of CE studies in our hospital included direct exposures; promoting use of last-image hold instead of direct exposure can reduce patient radiation dose. Our study demonstrates the importance of continuous quality assessment even in expert centers and we recommend that a Quality Standards checklist be used for all neonatal CE studies, possibly incorporated as part of a template report.

## Data Availability

The datasets generated during and/or analyzed during the current study are available from the corresponding author on reasonable request.
